# Prenatal cocaine exposure and self-reported behavioral adjustments from ages 12 to 21: environmental pathways

**DOI:** 10.1017/S0033291723002404

**Published:** 2023-08-24

**Authors:** Meeyoung O. Min, Jeffrey M. Albert, Sonia Minnes, June-Yung Kim, Sun-Kyung Kim, Lynn T. Singer

**Affiliations:** 1 College of Social Work, University of Utah, Salt Lake City, Utah, USA;; 2 Department of Population and Quantitative Health Sciences, School of Medicine, Case Western Reserve University, Cleveland, Ohio, USA;; 3 Jack, Joseph and Morton Mandel School of Applied Social Sciences, Case Western Reserve University, Cleveland, Ohio, USA;; 4 Department of Social Work, College of Nursing and Professional Disciplines, University of North Dakota, Grand Forks North Dakota, USA

**Keywords:** emerging adulthood, externalizing behaviors, internalizing behaviors, prenatal cocaine exposure, random-intercept cross-lagged panel model

## Abstract

**Background.:**

In a birth-cohort study, we followed offspring with prenatal cocaine exposure (PCE) to investigate longitudinal associations of PCE with self-reported behavioral adjustment from early adolescence to emerging adulthood (EA). Environmental pathways (family functioning, non-kinship care, maltreatment) were specified as potential mediators of PCE.

**Methods.:**

Participants were 372 (190 PCE; 47% male), primarily Black, low socioeconomic status, enrolled at birth. Internalizing and externalizing behaviors were assessed using Youth Self-Report at ages 12 and 15 and Adult Self-Report at age 21. Extended random-intercept cross-lagged panel modeling was used to account for potential bidirectional relationships between internalizing and externalizing behaviors over time, examining potential mediators.

**Results.:**

Adjusting for covariates, significant indirect effects were found for each mediator at different ages. For family functioning, these were both internalizing (*β* = 0.83, *p* = 0.04) and externalizing behaviors (*β* = 1.58, *p* = 0.02) at age 12 and externalizing behaviors at age 15 (*β* = 0.51, *p* = 0.03); for non-kinship care, externalizing behaviors at ages 12 (*β* = 0.63, *p* = 0.02) and 15 (*β* = 0.20, *p* = 0.03); and for maltreatment, both internalizing and externalizing behaviors at ages 15 (*β* = 0.64, *p* = 0.02 for internalizing; *β* = 0.50, *p* = 0.03 for externalizing) and 21 (*β* = 1.39, *p* = 0.01 for internalizing; *β* = 1.11, *p* = 0.01 for externalizing). Direct associations of PCE with internalizing and externalizing behaviors were not observed, nor cross-lagged relationships between internalizing and externalizing behaviors.

**Conclusions.:**

Negative associations of PCE with behavioral adjustment persist into EA via environmental pathways, specifying intervention points to disrupt adverse pathways toward healthy development.

## Introduction

Prenatal drug exposure remains a daunting public health problem that can affect child development and later educational and economic opportunities. The 2021 National Survey on Drug Use and Health (Substance Abuse and Mental Health Services Administration [SAMHSA], 2023) estimated that 7.7% of pregnant women used illicit drugs, with 0.2% using cocaine during the past month. When illicit drug use was combined with prior month’s use of tobacco or alcohol, the percentage was an alarming 19.6%, a rate that raises concerns about adverse outcomes in offspring across developmental stages.

Prenatal cocaine exposure (PCE) crosses the placental fetal blood–brain barrier and creates a cascade of teratologic effects via direct and indirect pathways (Mayes, 2002; Ross, Graham, Money, & Stanwood, 2015). These adverse effects on developmental outcomes have been demonstrated using brain imaging (Dow-Edwards et al., 2006) and measures of cognition across human and non-humans (Martin, Graham, McCarthy, Bhide, & Stanwood, 2016). Cocaine’s direct effect on the monoamine-rich frontal areas of the brain (McCarthy, Kabir, Bhide, & Kosofsky, 2014; Thompson, Levitt, & Stanwood, 2009) has led researchers to investigate evidence of disruptions of self-regulation and behavioral adjustment.

### PCE behavioral outcome studies

The association between PCE and self-regulatory behavior has been reported consistently among school-age children (for reviews, Ackerman, Riggins, & Black, 2010; Lambert & Bauer, 2012) and adolescents (for reviews, Buckingham-Howes, Berger, Scaletti, & Black, 2013; Min, Minnes, Yoon, & Singer, 2017b). Birth-cohort prospective studies have reported the relationship of PCE with child-reported oppositional defiant disorder and attention-deficit/hyperactivity disorder at 6 years of age (Linares et al., 2006), caregiver-reported aggressive behavior at 9 years (McLaughlin et al., 2011), child-reported depressive symptoms, caregiver-rated withdrawn behaviors, and teacher-rated anxious/depressed behaviors at 10 years (Richardson, Goldschmidt, Larkby, & Day, 2013), teacher- and caregiver-rated externalizing (i.e. aggressive and disruptive) behaviors at age 7, 9, 11, and 13 years (Bada et al., 2011), adolescent-reported externalizing behaviors at 12 and 15 years (Min et al., 2014a; Min, Minnes, Yoon, Short, & Singer, 2014b), and oppositional defiance disorder at age 17 (Kim et al., 2022). However, these studies typically examined externalizing and internalizing (i.e. depressive, anxious, withdrawn) behaviors separately, ignoring their well-documented co-occurrence (Bartels et al., 2018; Vergunst et al., 2023). Only one study to date has assessed behavioral outcomes through emerging adulthood (EA), reporting increased emotion regulation difficulties and conduct disorder at age 21 among offspring with PCE (Richardson, De Genna, Goldschmidt, Larkby, & Donovan, 2019). Studies examining specific environmental pathways by which PCE confers greater risk for behavioral problems across adolescence and EA have also been lacking.

### Environmental pathways linking PCE and behavioral adjustment in EA

In this study, we focused on three environmental pathways as mediating mechanisms of PCE that are likely to shape behavioral adjustment across adolescence and EA: (poor) family functioning, non-kinship care, and maltreatment. These pathways represent multiple underlying dimensions of adversity including deprivation, threat, and unpredictability (Ellis, Sheridan, Belsky, & McLaughlin, 2022), which are prevalent in families where caregivers misuse substances.

Family functioning comprises multiple interdependent dimensions, such as emotional bonding among family members (i.e. attachment), problem-solving and communication abilities (i.e. conflict resolution strategies), and adherence to family rules and cohesion (i.e. parental monitoring) (El-Sheikh & Buckhalt, 2003; Izzo, Baiocco, & Pistella, 2022). Lower quality attachment, conflict resolution, and parental monitoring may collectively represent a depriving caregiving environment. PCE is a strong antecedent to caregivers’ postnatal substance use (Elkington, Bauermeister, & Zimmerman, 2011; Singer et al., 2018), compromising their ability to promote a child’s healthy behavioral adjustment. We previously found that adolescents with PCE reported lower parental attachment and higher family conflict compared to those without PCE (Min et al., 2014b), making family functioning an important aspect of early development to evaluate in the context of PCE.

Non-kinship care, representing an aspect of environmental unpredictability, may be another critical pathway, as prenatal drug exposure is one of the most common precipitants of non-kinship caregiving placements for infants (Tung, Christian-Brandt, Langley, & Waterman, 2020). In our cohort with PCE, foster/adoptive care was associated with a better-quality home environment (Singer et al., 2004, 2008) and better language and adaptive functioning (Lewis et al., 2011; Singer, Powers, Kim, Minnes, & Min, 2023). However, the protective effects of non-kinship foster/adoptive placement were not generalized to behavioral outcomes, with non-kinship caregivers reporting more behavioral problems across developmental stages (Bada et al., 2008; Linares et al., 2006; McLaughlin et al., 2011) or no difference via adolescents’ self-report (Min et al., 2014a, 2014b).

Childhood maltreatment is also a critical pathway in behavioral adjustment (Godinet, Li, & Berg, 2014; Jonson-Reid, Kohl, & Drake, 2012) and represents salient threat and distinct environmental adversity. Adolescents with PCE reported higher rates of lifetime maltreatment, compared to a cohort of similar socio-demographic status without PCE (Kim et al., 2022; Singer et al., 2023). Childhood maltreatment was related to more negative coping behaviors to stress more strongly in adolescents with PCE than in adolescents without PCE in this cohort (Min, Minnes, Kim, Yoon, & Singer, 2017a).

### Current study

This study extends previous findings of PCE into EA by examining possible pathways in the longitudinal associations of PCE with self-reported behavioral adjustment from early adolescence to EA. Family functioning, non-kinship care, and maltreatment were specified as potential mediators of PCE. Given the established correlation between internalizing and externalizing problems and possible bidirectional relationships over time, we utilized a random-intercept cross-lagged panel model (RI-CLPM) to understand the nature of the co-development, controlling for confounders. Confounders included prenatal and post-natal environmental conditions correlated with PCE and shown to affect behavioral adjustment, such as greater exposure to alcohol, tobacco, and marijuana (Min et al., 2021a, 2021b; Richardson et al., 2019; Singer et al., 2021), a caregiver’s ongoing substance use (Elkington et al., 2011; Singer et al., 2018) and psychological distress (Min et al., 2018; Minnes et al., 2008), poorer quality of home environment (Lewis et al., 2013), violence exposure (Delaney-Black et al., 2002; Kobulsky, Minnes, Min, & Singer, 2016), and limited resources and support from school and neighborhood (Min, Minnes, Kim, Kim, & Singer, 2023; Tucker, Pollard, de la Haye, Kennedy, & Green, 2013). We also assessed sex and IQ as covariates given the sex difference in behavioral problems (Kim et al., 2022; Minnes et al., 2010) and a relationship between IQ and behavior problems (Pajer et al., 2008; Van Rest et al., 2020). We hypothesized that poorer family functioning, higher likelihood of non-kinship care, and/or maltreatment would mediate the relationship between PCE and greater internalizing and externalizing problems across adolescence and EA.

## Methods

### Sample and procedure

Participating offspring/emerging adults (*N* = 372) were recruited with their biologic mothers at birth (September 1994 to June 1996) from an urban hospital in the Midwest USA for a study of the developmental effects of PCE (Minnes et al., 2017; Singer et al., 2004). Immediately before/after childbirth, drug toxicology screenings were administered to 647 pregnant women at high risk for drug use due to lack of prenatal care, self-admitted drug use, behaviors suggesting intoxication, or a previous involvement with the Department of Children and Family Services. Of the screened, 155 refused to participate and 23 did not come to the enrollment visit; 54 were excluded due to maternal HIV-positive status, chronic medical or psychiatric illness, or low IQ, infants with Down syndrome, or fetal alcohol syndrome. Enrolled were 415 newborns and their mothers.

Maternal urine samples (*n* = 405) were collected in hospital within 24 h before/after delivery; infant meconium (*n* = 341) and urine samples (*n* = 24) were gathered shortly after infant birth. Biologic samples were analyzed for cocaine and other drug metabolites, including amphetamines, benzodiazepines, cannabinoids, cocaethylene, and opiates. Positive screens of maternal and infant urine, infant meconium, or maternal self-report determined 218 newborns as PCE. About 62% (136/218) of newborns with PCE were determined by both biological and maternal self-report measures, with 10% (*n* = 22) and 28% (*n* = 60) determined by either biologic assays or maternal self-report (Arendt, Singer, Minnes, & Salvator, 1999). Infants negative on all PCE indicators were classified as non-cocaine exposed (NCE). Both PCE and NCE groups were exposed to alcohol, tobacco, and marijuana. Examiners blind to substance exposure status conducted follow-up assessments at 1, 6, and 12 months and 2, 4, 6, 9, 10, 11, 12, 15, 17, and 21 years postpartum at the university-based research lab.

From the enrollment, 12 (9 PCE, 3 NCE) children died by the 12-year assessment from various medical conditions (e.g. sudden infant death syndrome, respiratory distress, pneumonia, etc.). We utilized data from 372 (190 PCE, 182 NCE) offspring with outcome data at any of three time points at ages 12 (*n* = 352), 15 (*n* = 358), or 21 (*n* = 299), representing 92% retention of the 403 living participants at age 12. Among 372 participants, 279 (75%) had all three assessments, 79 (21%) with two assessments, and 14 (3.76%) with one assessment. Compared to 372 participants, 31 non-participants (19 drop-out, 11 lost contact, 1 low intellectual function [IQ < 50]) were more likely to be White and prenatally exposed to tobacco. No other differences were observed. The Institutional Review Board of the participating hospital approved the study. Parental written informed consent was obtained at each visit prior to offspring age 18 years, with offspring assent beginning at age 9 and consent at age 21. With a Certificate of Confidentiality (DA-98–91) from the US Department of Health and Human Services, this study was exempt from the release of drug-related information. Monetary compensation, lunch, and/or transportation costs were provided to all participants.

### Measures

#### Prenatal cocaine and other substance exposures

At the newborn visit, birth mothers reported amount and frequency of drug use for the month prior to and for each trimester of pregnancy using a Timeline Follow Back method (Singer et al., 2002). For cocaine use, the number of rocks consumed and the amount of money expended on cocaine per day were recalled and computed to a standard unit of cocaine, equivalent to $20 worth of crack cocaine. The number of drinks of beer, wine, or hard liquor per week, with each drink equivalent to 0.5 oz. of absolute alcohol, was recorded. The number of tobacco cigarettes and marijuana joints smoked per day were collected. For each drug, frequency of use was rated on a Likert-type scale (0 = *not at all* to 7 = *daily use*) to indicate the average number of days per week of drug use, except for cigarettes computed as the number smoked per day. An average use across the pregnancy was calculated based on the frequency multiplied by the amount used per day. To assess recent (prior 30-day) postpartum, caregiver drug use, current caregivers updated the drug assessment at each follow-up visit.

#### Internalizing and externalizing behaviors

Internalizing and externalizing behaviors were assessed using the Youth Self-Report (YSR; Achenbach & Rescorla, 2001) at ages 12 and 15 and the Adult Self-Report (ASR; Achenbach & Rescorla, 2003) at age 21. The YSR and ASR yield two broadband scale scores: internalizing (*α* = 0.86–0.90) comprise items on anxious/depressed, withdrawn/depressed, and somatic problems; externalizing (*α* = 0.87–0.90) comprise items on aggressive and delinquent behaviors (and intrusive behaviors only in the ASR). Standardized *T* scores were used, with higher scores indicating greater symptoms.

#### Mediators

Parental attachment, parental monitoring, and family conflict were all assessed at age 12 using the Assessment of Liability and Exposure to Substance Use and Antisocial Behavior Scale (ALEXSA; Ridenour, Clark, & Cottler, 2009), an illustration-based, audio/computer-assisted self-report for children ages 9–12. The parental attachment scale (*α* = 0.80) assesses relational bonding with parents. The parental monitoring scale (*α* = 0.74) assesses whether parent(s) usually know(s) the youth’ activities, friendship, and whereabouts. For both parental attachment and monitoring scales (five items on a 4-point Likert scale), the average scores were used, with higher scores indicating greater levels of respective constructs (potential ranges = 0–3). The family conflict index (10 items; 1 = yes) assesses family conflict tactics (e.g. yelling, throwing objects) used to resolve family dispute. The total count was reverse coded to represent lower scores to be higher levels of family conflict. The *family functioning* latent variable (*α* = 0.65) was specified with the three indicators. *Childhood maltreatment* was self-reported at age 17 retrospectively using the Maltreatment module of Juvenile Victimization Questionnaire – Adult Retrospective (JVQ; Hamby, Finkelhor, Ormrod, & Turner, 2004). Any incidence of maltreatment, including physical abuse, psychological/emotional abuse, neglect, custodial interference/family abduction, was coded 1 (yes). Child placement was updated at each follow-up visit (1 = ever placed with *non-kinship* adoptive or foster care parents by age 12).

#### Potential confounders

Infant (sex, race, gestational age, birth weight, height, head circumference, Hobel scores) and maternal characteristics (age at delivery, marital status, education) were obtained from hospital birth records. Socioeconomic status (SES) was assessed using the Hollingshead Two-Factor Index (Hollingshead, 1971). Birth mothers’ receptive vocabulary was assessed via the Peabody Picture Vocabulary Test-Revised (Dunn & Dunn, 1981) and updated at subsequent assessments via its third edition (Dunn & Dunn, 1997). Maternal psychological distress was assessed using the Global Severity Index (*α* = 0.95), a summary scale of the Brief Symptom Inventory (Derogatis, 1992). Concurrent data on current caregivers’ vocabulary and psychological distress were updated at each visit.

Offspring Full-Scale IQ was assessed at age 11 using the Wechsler Intelligence Scales for Children-Fourth Edition (Wechsler, 2003). The quality of caregiving environment was assessed at age 12 via the caregiver-reported Home Observation of the Environment-Early Adolescent (*α* = 0.83; Caldwell & Bradley, 2003). Lifetime frequency of violence exposure (*α* = 0.75) was self-reported at age 12 using the ALEXSA. Ecological resources and support were assessed at age 12 using the External Assets subscale (*α* = 0.93) of the caregiver-reported Developmental Assets Profile (Scales & Leffert, 2004; Search Institute, 2005). Receipt of free school lunch (1 = yes) was recorded at age 15, and completion of high school (or equivalent) was self-reported at age 21.

### Data analyses

To examine mediation of PCE on externalizing and internalizing behaviors at ages 12, 15, and 21, an extended RI-CLPM (Hamaker, Kuiper, & Grasman, 2015) was used using Mplus 8.3. The RI-CLPM was extended with a path model with the three potential mediators linked to the repeatedly measured outcomes. This model allows for causal mediation analysis, utilizing the RI-CLPM structure to examine interconnections between externalizing and internalizing behaviors, while distinguishing within (time-varying) and between (time-invariant, trait-like) individual effects via random effects. Variables correlated (*p* < 0.15) with the outcomes for any time point were included in the model as covariates (prenatal exposure to alcohol, tobacco, or marijuana, offspring sex, IQ, HOME score, violence exposure, and external assets).

Maximum likelihood, allowing use of all available endogenous data for cases with values for all exogenous variables, was used to fit the models. Coefficients were tested using two-sided *z*-tests. Model fit was evaluated using the χ^2^ goodness-of-fit test, as well as the Comparative Fit Index (CFI), Tucker–Lewis Index (TLI), root mean square error of approximation (RMSEA), and standardized root mean square residual (SRMR) indices. Values ⩾0.95 for CFI and TLI, ⩽0.06 for RMSEA, and ⩽0.08 for SRMR indicate a good model fit (Hu & Bentler, 1999). Standardized estimates of coefficients were computed and interpreted.

Of the 372-participating offspring, missing covariates were found in 7% of the sample (*n* = 27), and the RI-CLPM analysis was based on 345 individuals with complete data on the included covariates. The analysis assumed missing completely at random for individuals and missing at random for outcomes. As the missing percentages were small, plausible violations of these assumptions were not expected to substantially affect results (Dong & Peng, 2013). When the analysis described above was re-done using multiple imputation as a sensitivity analysis, employing a Markov Chain Monte Carlo algorithm and variance covariance model to obtain 30 completed data sets via the Bayesian estimation approach, no substantial differences were noted in all path coefficients and indirect effects using Rubin (1987)’s approach. Thus, we presented findings based on 345 individuals with complete data on the included covariates.

Indirect effects of PCE were estimated using the products of coefficients approach and tested using *z*-tests based on delta method estimates of standard errors (VanderWeele, 2015). Proportions mediated by specific indirect paths were calculated to illustrate the relative strength of each mediator on behavioral outcomes at different ages. Statistical significance was set at *p* < 0.05.

## Results

### Sample characteristics

The birth mothers of infants with PCE were older, less educated, less likely to be married, had greater psychological distress and lower vocabulary scores, and reported greater use of tobacco, alcohol, and marijuana over their pregnancy than mothers of infants with NCE (Table 1). No difference was found in caregiver characteristics at age 12, except that caregivers of children with PCE smoked more cigarettes in the past 30 days than caregivers of NCE offspring.

Compared to those with NCE, infants with PCE had shorter gestational ages and lower birth weight, length, and head circumference (Table 2). The PCE group reported lower parental attachment and higher family conflict; were more likely to be placed in non-kinship foster or adoptive care by age 12 and to report maltreatment. The PCE group reported greater externalizing behaviors at ages 12 and 15 than the NCE group, but no difference at age 21. No group differences were found in internalizing behaviors across all three assessments. Those with PCE were less likely to complete high school than the NCE group. Table 3 summarizes bivariate correlations among key observed variables included in the extended RI-CLPM.

### Model estimation

**Figure 1.**
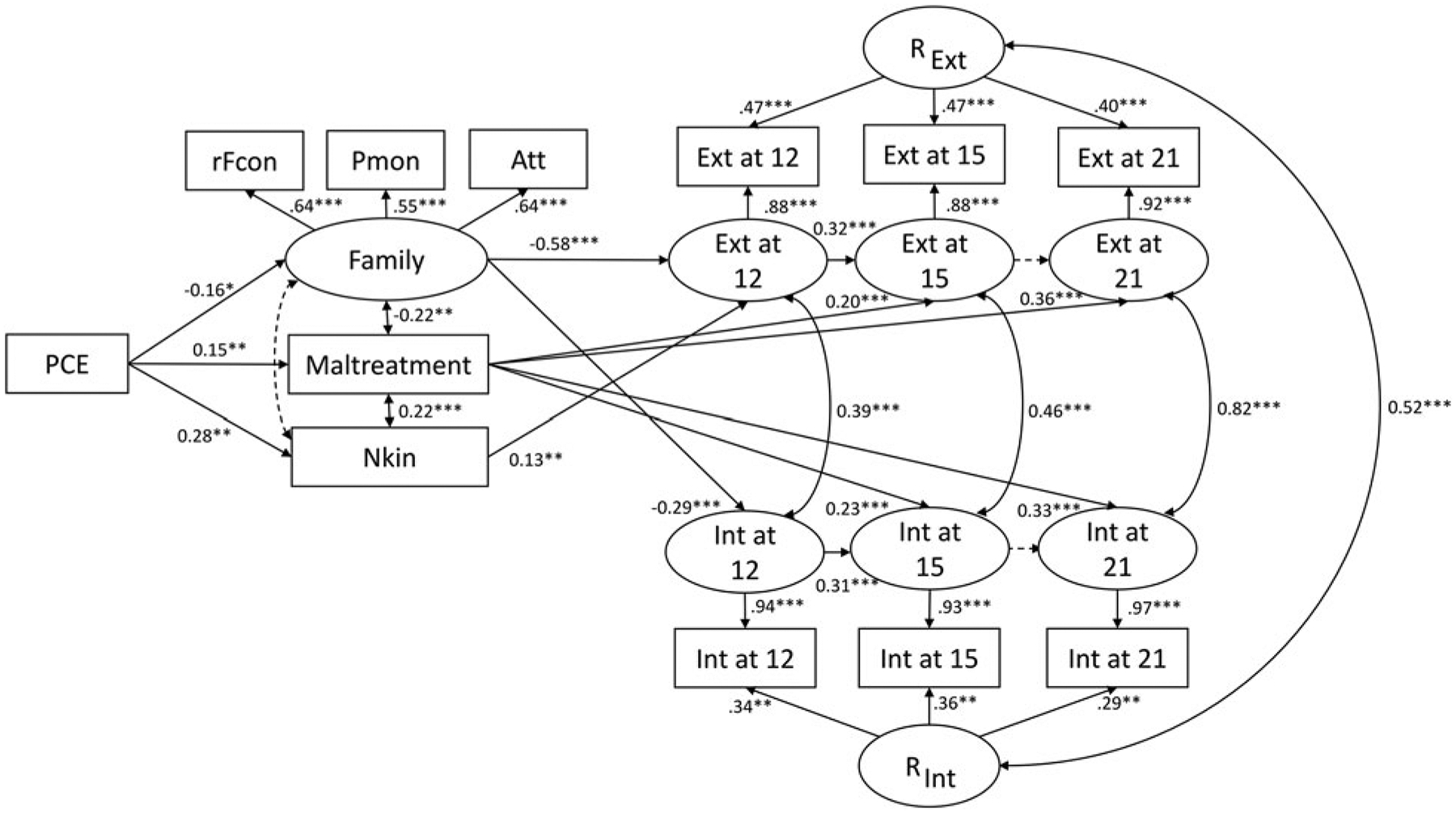
Extended random-intercept cross-lagged panel model of the association between prenatal cocaine exposure and externalizing and internalizing behaviors from early adolescence to emerging adulthood. χ^2^ (102) = 180.18, *p* < 0.001, CFI = 0.936, TLI = 0.910, RMSEA = 0.047 (90% CI 0.036–0.058), SRMR = 0.068. Rectangles indicate observed variables, and ovals represent latent constructs. All path coefficients are standardized; single-arrowed lines represent path coefficients, and double-arrowed lines represent correlations. Solid lines indicate statistically significant (*p* ⩽ 0.05) paths, and dotted lines indicate non-significant (*p* > 0.05) paths. Only significant path coefficients are shown; covariates and errors associated with the measurement model are not displayed for parsimony. Att, parental attachment; Ext, externalizing behaviors; Family, family functioning; Int, internalizing behaviors; Nkin, ever in non-kinship care by age 12; PCE, prenatal cocaine exposure; rFcon, family conflict, reverse coded; Pmon, parental monitoring; R_Ext_, latent stability/trait of externalizing behaviors over-time; R_Int_, latent stability/trait of internalizing behaviors over-time. Estimates are adjusted for prenatal exposure to alcohol (on Nkin and Int at 21), tobacco (on Nkin and Int at 21), and marijuana (on Int at 12), biological sex (on Int at 12 and 15 and Ext at 15), violence exposure (on Int and Ext at 12), IQ (on Int at 12), and external assets (on Ext at 12, 15, and 21). *p ⩽ 0.05, **p ⩽ *0*.01, ***p ⩽ 0.001.

Figure 1 presents the final model produced from iterative model-fitting procedures. The initial, saturated model included (1) paths from PCE to all three mediators and the repeated measures of externalizing and internalizing behaviors at three ages; (2) paths from the covariates to all three mediators and the repeated measures of externalizing and internalizing behaviors; (3) correlations between the three mediators; (4) the lag-one autoregressive and cross-lagged paths; (5) correlations between internalizing and externalizing behaviors within the same assessment point; and (6) correlations between all exogenous (i.e. PCE and all covariates) variables. This initial model produced an acceptable fit, χ^2^ (47) = 130.94, *p* < 0.001, CFI = 0.931, TLI = 0.775, RMSEA = 0.072 (90% CI 0.057–0.087), SRMR = 0.059. To achieve a more parsimonious model, path coefficients with significance level of *p* ⩾ 0.15 were set to zero (Hosmer, Lemeshow, & Sturdivant, 2013), χ^2^ (102) = 180.18, *p* < 0.001, CFI = 0.936, TLI = 0.910, RMSEA = 0.047 (90% CI 0.036–0.058), SRMR = 0.068, yielding insignificant difference in model fit, Δχ^2^ (55) = 49.24, *p* = 0.69. This reduced model was accepted as the final model (Fig. 1).

Adjusting for relevant covariates, PCE was related to poorer family functioning at age 12 (*β* = −0.16, S.E. = 0.065, *p* = 0.01), which in turn was related to greater internalizing (*β* = −0.29, S.E. = 0.073, *p* < 0.001; indirect effect of PCE, *β* = 0.83, S.E. = 0.39, *p* = 0.036) and externalizing (*β* = −0.58, S.E. = 0.063, *p* < 0.001; indirect effect of PCE, *β* = 1.58, S.E. = 0.65, *p* = 0.016) behaviors at age 12. Greater externalizing behaviors at age 12 were related to greater externalizing behaviors at age 15 (*β* = 0.32, S.E. = 0.055, *p* < 0.001; indirect effect of PCE via family functioning, *β* = 0.51, S.E. = 0.23, *p* = 0.028). Although greater internalizing behaviors at age 12 were related to greater internalizing behaviors at age 15 (*β* = 0.31, S.E. = 0.069, *p* < 0.001), no significant indirect effect of PCE was observed via family functioning on internalizing behaviors at age 15 (*β* = 0.24, S.E. = 0.13, *p* = 0.062). PCE was also related to a higher likelihood of non-kinship placement (*β* = 0.28, S.E. = 0.053, *p* < 0.001). Non-kinship placement was related to more externalizing behaviors at age 12 (*β* = 0.13, S.E. = 0.049, *p* = 0.007; indirect effect of PCE, *β* = 0.63, S.E. = 0.26, *p* = 0.016), which contributed to greater externalizing behaviors at age 15 (indirect effect of PCE, *β* = 0.20, S.E. = 0.09, *p* = 0.027). PCE was related to a greater exposure to maltreatment (*β* = 0.15, S.E. = 0.054, *p* = 0.005), which in turn was related to greater internalizing behaviors at ages 15 (*β* = 0.23, S.E. = 0.056, *p* < 0.001; indirect effect of PCE, *β* = 0.64, S.E. = 0.27, *p* = 0.019) and 21 (*β* = 0.33, S.E. = 0.056, *p* < 0.001; indirect effect of PCE, *β* = 1.39, S.E. = 0.53, *p* = 0.011) and externalizing behavior at ages 15 (*β* = 0.20, S.E. = 0.055, *p* < 0.001; indirect effect of PCE, *β* = 0.50, S.E. = 0.23, *p* = 0.029) and 21 (*β* = 0.36, S.E. = 0.056, *p* < 0.001; indirect effect of PCE, *β* = 1.11, S.E. = 0.44, *p* = 0.012).

Direct associations of PCE with internalizing and externalizing behaviors were not observed, nor auto-regressive paths of both externalizing and internalizing behaviors from ages 15 to 21. Although internalizing and externalizing behaviors were associated within offspring (i.e. correlation within time) across all assessments (ranged *r* = 0.39 at age 12 to *r* = 0.82 at age 21; Fig. 1), no cross-lagged paths between internalizing and externalizing behaviors were significant, with time-invariant, trait-like characteristics shared by both internalizing (R_Int_) and externalizing (R_Ext_) domains being partialled out. These two latent trait factors were substantially correlated (*r* = 0.52). Table 4 summarizes significant indirect effects of PCE via these three mediators at age 12, 15, and 21, along with mediated proportions (%) via each mediator.

Regarding covariates, prenatal exposure to alcohol (*β* = 0.14, S.E. = 0.051, *p* = 0.008) and tobacco (*β* = 0.1, S.E. = 0.053, *p* = 0.045) were related to greater likelihood of non-kinship placement. Greater violence exposure was related to both internalizing (*β* = 0.20, S.E. = 0.057, *p* < 0.001) and externalizing behaviors (*β* = 0.24, S.E. = 0.054, *p* < 0.001) at age 12. Higher IQ was related to fewer internalizing behaviors at age 12 (*β* = −0.21, S.E. = 0.048, *p* < 0.001). Greater external assets were related to fewer externalizing behaviors across all three assessments (*β* = −0.18, S.E. = 0.048, *p* < 0.001 at age 12; *β* = −0.12, S.E. = 0.049, *p* = 0.013 at age 15; *β* = −0.13, S.E. = 0.038, *p* = 0.001). Males reported higher internalizing behaviors at age 12 (*β* = 0.12, S.E. = 0.049, *p* = 0.013), yet fewer externalizing behaviors at age 15 (*β* = −0.16, S.E. = 0.051, *p* = 0.002) than females.

## Discussion

This study demonstrated that PCE-related behavioral problems persist into EA via multiple environmental pathways. PCE was related to poorer family functioning, a greater exposure to maltreatment, and a greater likelihood of placement in non-kinship care in childhood, each of which contributed to elevated externalizing and internalizing behaviors across adolescence and EA, supporting the long-lasting, unfolding influence of PCE channeled through environmental factors (Mayes, 2002). The family environment and non-kinship placement appeared to be more salient at ages 12 and 15, whereas maltreatment was salient at ages 15 and 21. Although these specific pathways have been previously reported to contribute to behavioral adjustment, they have seldom been examined simultaneously. Given the clustered nature of multiple adversities present in offspring with PCE, our simultaneous analysis of multiple environmental pathways suggests specific points of intervention that may better support healthy development.

Our study observed four noteworthy findings in the longitudinal relationship between internalizing and externalizing behaviors. First, no cross-lagged relationship was observed, suggesting that internalizing and externalizing problems are not causally related to each other. Our study supports the shared-risk hypothesis that the co-development of internalizing and externalizing problems is due to shared risk factors related to both externalizing and internalizing behaviors (Angold, Costello, & Erkanli, 1999). PCE, mediated by family functioning, non-kinship care, and maltreatment, is a common correlate to explain such co-development, at least partially. Second, the time-invariant component of internalizing and externalizing problems was substantially correlated, indicating that co-development of these behavioral problems is partly attributable to stable, trait-like characteristics related to both externalizing and internalizing problems. Previous studies suggested that deficits in executive function in early childhood could be a common antecedent associated with greater problems in both internalizing and externalizing behaviors (e.g. Hughes & Ensor, 2011; Oh, Greenberg, Willoughby, & The Family Life Project Key Investigators 2020). Given the poorer executive function noted in children with PCE (Minnes et al., 2014; Noland et al., 2003), future studies examining interrelationships among PCE, executive function, and behavioral adjustment, along with the three environmental pathways identified in this study, will enhance our understanding of PCE-associated problems across developmental stages. Third, there were no significant auto-regressive paths in both internalizing and externalizing behaviors from 15 to 21 years, demonstrating a low degree of stability. Although this low stability could be due to the long assessment interval, it may also be attributed to the nature of the observed period, as ages 15–21 represent a volatile developmental period of opportunities for both enrichment and risk experimentation, subject to escalating both adaptive and maladaptive adjustment (Arnett, 2007; Cicchetti & Rogosch, 2002; Min, Yoon, Minnes, Ridenour, & Singer, 2019). Lastly, the correlation between internalizing and externalizing behaviors is much higher on the ASR at 21 years than the YSR at 12 and 15 years, mirroring the lifetime prevalence of multimorbid disorder increased with age: 14.2% at ages 13–14, 29.9% at ages 15–16 (Merikangas et al., 2010), and 33.9% at ages 18–29 (Kessler et al., 2005). It may also reflect the increasing prevalence of many mental disorders from childhood to young adulthood (Merikangas et al., 2010).

These findings should be interpreted considering limitations. First, we relied on offspring self-report on internalizing and externalizing problems, subject to offspring ability and willingness to assess and reflect on their own problems. Second, we operationalized non-kinship care as the experience of non-kinship care during the first 12 years of life, but the lack of explicit details might not fully account for the experience and its relationships with behavioral adjustment. The account of maltreatment was obtained retrospectively, prone to recall error and social desirability bias. Third, given the relatively small sample size and complexity of the model, separate models for males *v*. females were not tested, an important consideration for future studies. Fourth, although we controlled multiple confounders of PCE, our observational design does not dismiss that the indirect association of PCE with behavioral adjustment could be a function of unmeasured confounders of PCE. Lastly, generalizability of the findings may be limited to urban, predominantly African-American individuals of low SES whose mothers were assessed for prenatal drug use at delivery.

Strengths of the study include its longitudinal prospective design with multiple follow-ups from birth, use of multiple methods including biological markers to identify PCE, desirable retention rates, and the careful assessments of numerous environmental variables to characterize the developmental context. In addition, our novel analytical approach to separate between- and within-person effects in examining reciprocal processes between internalizing and externalizing problems over time clarified their apparent bidirectional relationships. Our study indicates that PCE increases risk for behavioral problems from ages 12 to 21 through poor family functioning, non-kinship care placement, or/and maltreatment, underscoring the critical implication of reducing substance use among pregnant women to improve next generation’s mental wellbeing. Continued studies into later adulthood could expand our understanding of how PCE may contribute to behavioral, functional, and health outcomes over time.

## Figures and Tables

**Table 1.** Maternal and caregiver characteristics by PCE status

	PCE (*n* = 190)		NCE (*n* = 182)		
	*M*	s.d.	*M*	s.d.	*p*
*Biological mother*					
African-American, *n* (%)	157	(82.6)	148	(81.3)	0.79
Low socioeconomic status, *n* (%)	185	(97.9)	178	(97.8)	0.99
Age at delivery	29.69	4.99	25.46	4.73	<0.001
Years of education	11.56	1.66	11.95	1.39	0.01
No high school diploma, *n* (%)	92	(48.4)	59	(32.4)	0.002
Married, *n* (%)	15	(7.9)	29	(15.9)	0.02
BSI Global Severity Index	0.83	0.75	0.50	0.53	<0.001
PPVT Standard Score	73.47	14.29	77.76	14.71	0.005
Substance use during pregnancy					
Alcohol drinks per week	9.79	17.48	1.42	4.60	<0.001
Cigarettes per day	11.65	11.18	5.43	7.18	<0.001
Marijuana joints per week	1.33	3.45	0.87	3.52	<0.001
Cocaine, units per week	22.46	37.80	-	-	-
*Caregiver at child age 12*					
HOME score	47.83	6.96	49.04	6.21	0.08
BSI Global Severity Index	0.36	0.45	0.36	0.49	0.90
PPVT Standard Score	79.32	14.69	79.58	15.65	0.87
Substance use in the past 30 days^a^					
Alcohol drinks per week	1.42	3.67	1.72	5.50	0.97
Cigarettes per day	5.43	7.56	3.86	6.77	0.01
Marijuana joints per week	0.87	7.05	0.10	1.07	0.16

BSI, Brief Symptom Inventory; HOME, Home Observation for Measurement of the Environment; NCE, non-cocaine exposed; PCE, prenatal cocaine exposed; PPVT, Peabody Picture Vocabulary Test.

a No caregivers reported cocaine use in the past 30 days.

**Table 2.** Offspring characteristics by PCE status

	PCE (*n* = 190)		NCE (*n* = 182)		
	*M*	s.d.	*M*	s.d.	*p*
*At birth*					
Gestational age (GA), weeks	37.81	2.86	38.47	2.87	0.03
Prematurity (<37 GA), *n* (%)	55	(29.0)	35	(19.2)	0.03
Birth weight, g^a^	2710.2	648.2	3101.6	698.0	<0.001
Birth length, cm^a^	47.33	3.96	49.13	3.74	<0.001
Head circumference, cm^a^	32.29	2.15	33.48	2.38	<0.001
Hobel Neonatal Risk Score	7.55	16.53	5.83	15.83	0.31
Male, *n* (%)	85	(44.8)	89	(48.9)	0.47
African-American, *n* (%)	156	(82.1)	147	(80.8)	0.79
*At age 11*					
WISC-IV Full-Scale IQ	84.70	11.79	86.41	14.70	0.22
*At age 12*					
Violence exposure	0.62	0.74	0.57	0.78	0.54
DAP external assets	21.22	5.14	21.43	4.68	0.68
Parental monitoring	2.42	0.64	2.48	0.59	0.39
Parental attachment	2.09	0.68	2.27	0.61	0.01
Family conflict^b^	6.78	2.55	7.40	2.37	0.02
Ever in non-kinship foster/adoptive care by age 12, *n* (%)	76	(40.0)	13	(7.1)	<0.001
YSR internalizing behaviors	53.38	9.96	53.30	9.34	0.94
YSR externalizing behaviors	50.46	9.96	47.47	9.81	0.004
*At age 15*					
Receiving free school lunch, *n* (%)	149	(83.7)	143	(84.6)	0.82
YSR internalizing behaviors	54.74	9.80	54.34	9.18	0.69
YSR externalizing behaviors	52.97	10.47	50.63	9.42	0.03
*At age 17*					
Maltreatment, *n* (%)	53	(31.0)	31	(18.0)	0.006
*At age 21*					
High school completion^c^, *n* (%)	118	(74.2)	133	(85.5)	0.01
ASR internalizing behaviors	48.95	14.15	46.69	12.83	0.15
ASR externalizing behaviors	47.62	11.42	45.86	11.16	0.18

ASR, Adult Self-Report; DAP, Developmental Asset Profile; NCE, non-cocaine exposed; PCE, prenatal cocaine exposed; WISC-IV, Wechsler Intelligence Scales for Children-Fourth Edition; YSR, Youth Self-Report.

a Adjusted for gestational age.

b Reverse coded; higher score indicates lower levels of family conflict.

c Assessed with a self-report, referring to either graduation from high school or passing the General Educational Development test.

**Table 3.** Correlations among key observed variables in the extended random-intercept cross-lagged panel model

	Covariates									Mediators					Behavioral adjustment					
	1	2	3	4	5	6	7	8	9	10	11	12	13	14	15	16	17	18	19	20
1. PCE	-	**0.49**	**0.50**	**0.22**	−0.04	−0.06	−0.09	0.03	−0.02	−**0.14**	−0.05	−**0.13**	**0.38**	**0.15**	0.00	**0.15**	0.02	**0.12**	0.08	0.08
2. Pre. alc. exp.^a^		-	**0.43**	**0.14**	−0.05	−0.06	−0.06	0.01	0.02	−**0.11**	−0.06	−**0.13**	**0.28**	0.03	0.05	**0.12**	0.04	0.08	−0.01	0.00
3. Pre. tob. exp^a^			-	**0.20**	−0.05	−0.03	−0.09	0.01	−0.02	−0.08	0.01	−0.10	**0.26**	0.05	−0.05	0.08	0.02	0.06	0.09	0.08
4. Pre. mar. exp.^a^				-	0.03	0.06	−0.05	−0.05	−0.07	−0.01	0.06	−0.05	0.03	0.10	0.02	0.02	0.01	0.04	0.11	0.06
5. Male					-	−0.08	−0.10	**0.11**	−**0.15**	−0.04	−**0.13**	0.08	−0.01	−0.05	**0.14**	0.05	< 0.01	−0.08	−0.09	0.03
6. Full-Sscale IQ						-	**0.15**	−**0.17**	**0.18**	−0.01	**0.20**	−0.01	−**0.14**	−**0.11**	−**0.27**	−**0.11**	−**0.19**	−0.10	−0.03	−0.06
7. HOME score							-	−0.07	**0.38**	**0.13**	0.03	−0.03	0.07	−0.08	−0.02	−**0.11**	−0.04	−**0.12**	<0.01	−0.02
8. Violence exp.								-	−0.10	−**0.19**	−**0.26**	−**0.27**	0.00	**0.20**	**0.32**	**0.38**	**0.14**	**0.22**	0.05	0.10
9. DAP external assets									-	**0.13**	0.09	0.10	0.02	−0.03	−0.10	−**0.23**	−**0.12**	−**0.20**	−0.03	−**0.14**
**Mediators**																				
10. Parental attachment										-	**0.39**	**0.45**	−0.04	−**0.12**	−**0.18**	−**0.35**	−**0.13**	−**0.16**	−0.10	−0.07
11. Parental monitoring											-	**0.32**	−0.07	−**0.15**	−**0.25**	−**0.37**	−0.03	−**0.15**	0.02	−0.07
12. Family conflict^b^												-	−0.10	−0.09	−**0.23**	−**0.43**	−**0.12**	−**0.17**	−**0.18**	−**0.14**
13. Ever in non-kinship care^c^													-	**0.24**	**0.12**	**0.21**	**0.13**	0.09	0.10	**0.14**
14. Maltreatment														-	**0.20**	**0.23**	**0.25**	**0.26**	**0.30**	**0.28**
**Behavioral adjustment**																				
15. Internalizing at age 12															-	**0.54**	**0.42**	**0.25**	**0.19**	**0.19**
16. Externalizing at age 12																-	**0.28**	**0.53**	**0.17**	**0.31**
17. Internalizing at age 15																	-	**0.54**	**0.34**	**0.29**
18. Externalizing at age 15																		-	**0.23**	**0.36**
19. Internalizing at age 21																			-	**0.80**
20. Externalizing at age 21																				-

*Note*. Bold indicates significance at *p* < 0.05; DAP, Developmental Asset Profile; HOME, Home Observation for Measurement of the Environment; PCE, prenatal cocaine exposure; Pre. alc. exp., prenatal alcohol exposure; Pre. tob. exp., prenatal tobacco exposure; Pre. mar. exp., prenatal marijuana exposure.

a Log-transformed.

b Reverse coded.

c By age 12.

**Table 4.** Indirect effects of PCE on behavioral adjustments from ages 12 to 21

	*β*	s.e.	*p*	*Mediated* %*
PCE → 12-year internalizing				
Via family functioning	0.83	0.39	0.036	81
PCE → 12-year externalizing				
Via family functioning	1.58	0.65	0.016	71
Via non-kinship placement	0.63	0.26	0.016	29
PCE → 15-year internalizing				
Via family functioning	0.24	0.13	0.062	28
Via maltreatment	0.64	0.27	0.019	72
PCE → 15-year externalizing				
Via family functioning → 12-year externalizing	0.51	0.23	0.028	42
Via non-kinship placement → 12-year externalizing	0.20	0.09	0.027	17
Via maltreatment	0.50	0.23	0.029	41
PCE → 21-year internalizing				
Via maltreatment	1.39	0.53	0.011	98
PCE → 21-year externalizing				
Via maltreatment	1.11	0.44	0.012	100

PCE, prenatal cocaine exposure.

* Calculated the specific indirect effect divided by the sum of all indirect effects. For example, for 12-year externalizing behaviors, family functioning mediated 71% [= (1.58/(1.58 + 0.63))×100] of the indirect effect of PCE, while non-kinship placement mediated 29% [= (0.63/(1.58 + 0.63))×100] of the indirect effect of PCE.
